# Injection of an improperly stored proprotein convertase subtilisin/kexin type 9 monoclonal antibody in a patient with secondary dyslipidemia from nephrotic syndrome: a case report

**DOI:** 10.1186/s13256-023-03804-5

**Published:** 2023-03-11

**Authors:** Tanawan Kongmalai, Nalinee Chuanchaiyakul, Yuttana Srinoulprasert, Nuntakorn Thongtang

**Affiliations:** 1 Division of Endocrinology and Metabolism, Department of Medicine, Faculty of Medicine Siriraj Hospital, Mahidol University, Bangkok, Thailand; 2 Department of Immunology, Faculty of Medicine Siriraj Hospital, Mahidol University, Bangkok, Thailand

**Keywords:** Nephrotic syndrome, Dyslipidemia, PCSK9 monoclonal antibody, PCSK9 storage, Temperature, Case report

## Abstract

**Background:**

Elevated plasma cholesterol and/or plasma triglyceride levels in nephrotic syndrome patients are the result of impaired lipoprotein clearance and a compensatory increase in hepatic lipoprotein synthesis. Plasma proprotein convertase subtilisin/kexin type 9 levels directly correlate to the amount of proteinuria in nephrotic syndrome patients. Proprotein convertase subtilisin/kexin type 9 monoclonal antibody has been used to treat dyslipidemia in some refractory nephrotic syndrome cases. As a therapeutic protein, proprotein convertase subtilisin/kexin type 9 monoclonal antibody simply deteriorates if stored in inappropriate temperatures or conditions.

**Case presentation:**

In this article, we present the case of a 16-year-old Thai female with severe combined dyslipidemia secondary to refractory nephrotic syndrome. She received proprotein convertase subtilisin/kexin type 9 monoclonal antibody (alirocumab) treatment. However, the drugs were mistakenly frozen in a freezer for up to 17 hours before being stored at 4 °C. After using two frozen devices, serum total cholesterol, free proprotein convertase subtilisin/kexin type 9, and lipoprotein(a) significantly decreased. Nonetheless, the patient developed a skin rash 2 weeks after the second injection and the lesion spontaneously resolved without any treatment approximately 1 month later.

**Conclusions:**

The effectiveness of proprotein convertase subtilisin/kexin type 9 monoclonal antibody seems to be stable after being stored under freeze–thaw conditions. However, improperly stored drugs should be discarded to avoid any potential undesirable side effects.

## Background

Nephrotic syndrome (NS) is one of the most common causes of secondary hyperlipidemia in children and adults. In the plasma of NS patients, lipoproteins are elevated, including intermediate-density lipoprotein (IDL), very low-density lipoprotein (VLDL), and low-density lipoprotein (LDL). NS patients have downregulation of hepatic lipase and lipoprotein lipase activities, and have high plasma proprotein convertase subtilisin/kexin type 9 (PCSK9) levels. PCSK9 breaks down the LDL receptor, and thus reduces LDL clearance and elevates plasma LDL-cholesterol (LDL-C) levels [1]. As a result, patients with refractory NS exposed to long durations of high plasma LDL-C are more likely to develop atherosclerosis, associated with cardiovascular complications, progressive kidney disease, and premature death. Currently, there is no specific recommendation for management of dyslipidemia in patients with refractory NS. However, a substantial percentage of patients achieve plasma lipid goals using a variety of therapies, including control of diet, use of statins, fibrates, bile acid sequestrants, nicotinic acid, ezetimibe, and LDL-apheresis, in addition to management of NS.

Plasma levels of PCSK9 directly correlate to the amount of proteinuria in patients with NS [1]. Remission in NS patients is associated with a decrease in plasma cholesterol levels and free PCSK9 [2]. In 2017, Awanami *et al*. reported successful treatment of a patient with refractory nephrotic syndrome with PCSK9 inhibitors [3]. Nowadays, PCSK9 monoclonal antibody (mAb) has received attention as a lipid-lowering strategy in NS patients due to its effectiveness in treating dyslipidemia. Nevertheless, its novelty and high cost are the main practical barriers. It is also possible that patients and medical professionals are not familiar with this new medication and are unaware of how to properly store the drugs.

PCSK9 mAb is protein, and thus prone to a variety of degradation pathways, especially if stored in inappropriate temperatures or conditions [4, 5]. Hence, manufacturers recommend keeping unopened drugs between 2 °C and 8 °C or at room temperature not exceeding 25 °C for a maximum of 30 days to get the best therapeutic effect. The manufacturer also recommends immediately abandoning these expensive agents if they are not stored in proper conditions. Despite an emphasis on proper drug storage, unexpected errors during storage can occur. We previously reported a decrease in PCSK9 inhibitory activity of PCSK9 mAb stored in room temperature conditions in vitro [5]. However, the effect of injecting inappropriately stored drugs in humans has never been reported. In this study, we present the case of a young female who suffered from severe combined dyslipidemia secondary to refractory nephrotic syndrome. She was treated with PCSK9 mAb. However, the drug was stored in a freezer before being moved to the refrigerator compartment again. Two freeze–thawed devices were used as the patient refused to have the drugs discarded despite being informed of the unknown effects of using improperly stored drugs. Her clinical and laboratory investigations, which included plasma free PCSK9 and lipoprotein(a) [Lp(a)], were carefully monitored. According to our comprehensive review, this is the first report of a patient injected with PCSK9 mAb stored improperly in a freezer.

## Case presentation

A 16-year-old Thai female was diagnosed with steroid-resistant nephrotic syndrome with secondary combined dyslipidemia at the age of 3 years. She had no history of cardiovascular diseases or pancreatitis, nor family history of dyslipidemia or diabetes mellitus. A physical examination revealed no arcus juvenilis, tendon xanthoma, or xanthelasma. When the patient was 3 years old, she presented with generalized edema. Laboratory findings revealed severe proteinuria, severe hypoalbuminemia [albumin 12 g/L (1.2 g/dL)] and hypercholesterolemia [total cholesterol level of 23.4 mmol/L (904 mg/dL)]. Based on her clinical presentations and laboratory findings, nephrotic syndrome was diagnosed. A kidney biopsy demonstrated focal segmental glomerulonephritis (FSGS). After confirming the diagnosis, she was treated with prednisolone 2 mg/kg/day for 1 month, leading to improved urine protein. Prednisolone was gradually tapered off, but the patient experienced worsening generalized edema and severe proteinuria. Therefore, oral cyclophosphamide was initiated along with an increased dosage of prednisolone. Unfortunately, she experienced multiple episodes of nephrotic syndrome relapse. Many immunosuppressive agents, including mycophenolate mofetil (MMF), tacrolimus, and rituximab were challenged one by one to control her disease.

Three years ago, despite the use of various multiple immunosuppressive medicines, the patient still had active nephrotic syndrome. Her total plasma cholesterol and triglyceride levels were continuously elevated. Meanwhile, her combined dyslipidemia was treated with simvastatin 10 mg/day and gemfibrozil 600 mg/day by a previous doctor. Subsequently, the statin was changed to atorvastatin to prevent muscle side effects. However, her plasma total cholesterol levels were between 13.1 and 19.5 mmol/L (504–752 mg/dL), plasma triglyceride levels between 1.2 and 15.8 mmol/L (106–1399 mg/dL), and plasma LDL-C levels between 9.4 and 16.0 mmol/L (362–620 mg/dL) over the past 13 years. As a result, 75 mg alirocumab every 2 weeks was prescribed in addition to statin and gemfibrozil, and her plasma LDL-C levels decreased from 447 to 328 mg/dL (−26.6%) after a single dose of properly stored 75 mg/day alirocumab, and it continuously decreased afterward (Table 1).

**Table 1:** Lipid profile, severity, and medications for treating nephrotic syndrome before and after PCSK9 injection

Date	Before frozen alirocumab injection						After frozen alirocumab injection				
	22/3/18	5/4/18	2/6/18	4/10/18	27/11/18	25/12/18	5/2/19	14/2/19	21/3/19	25/4/19	4/7/19
Free PCSK9 (ng/mL)	–	–	–	–	–	–	1043.3	943.1	811.5	810.4	778.2
TC (mg/dL)*	621	618	752	504	570	466	303	317	279	520	191
TG (mg/dL)*	1399	106	530	255	303	207	225	329	336	315	194
HDL-C (mg/dL)*	44	36	55	107	62	97	127	81	71	67	72
LDL-C (mg/dL)*	362	380	620	429	447	328	193	236	207	225	100
Lipoprotein (a) (nmol/L)	–	–	–	–	–	–	338.1	315.3	173.3	187.2	173.4
Albumin (g/dL)*	1.6	1.8	–	2.5	1.9	–	3.2	3.7	–	–	1.9
UPCR (mg/gCr)	25.8	14.5	18.9	10.8	15.8	–	3.6	3.8	2.2	6.3	2.5
Simvastatin (mg/day)	–			10	10	10	10	10	10	20	10
Gemfibrozil (mg/day)	Fenofibrate 200 mg/day	300	600	600	600	600	600	600	600	300	600
Prednisolone (mg/day)	–	60	50	20	15	20	35	30	20	15	10
Immunosuppressive agents	–		MMF 1000 mg/day	MMF 2000 mg/day	MMF 2000 mg/day	MMF 2000 mg/day	Cyclosporin 150 mg/day	Cyclosporin 200 mg/day		Cyclosporin 50 mg/day	Cyclosporin 100 mg/day
Alirocumab					75 mg sc	75 mg sc	Frozen alirocumab 75 mg was injected on 8/1/19 and 22/1/19				

*TC* total cholesterol, *TG* triglyceride, *LDL-C* low-density lipoprotein cholesterol, *HDL-C* high-density lipoprotein cholesterol, *MMF* mycophenolate mofetil, *Cr* creatinine, *UPCR* urine protein urine creatinine ratio, *sc* subcutaneous

*Conversion of conventional units to international units: TC or HDL-C or LDL-C X 0.0259 (mmol/l), TG X 0.0113(mmol/l), albumin X 10 (g/L)

Two days after receiving the second dose of alirocumab, she was hospitalized for acute diarrhea and acute kidney injury. Her parents requested medical staff to store PCSK9 mAb at the hospital after admission. Since healthcare professionals were unfamiliar with this new medicine and had never been trained on how to store it properly, alirocumab was put in a freezer from 3 pm to 8 am the next day (a total of 17 hours). The frozen drug was then moved to the refrigerator compartment (4°C), and after 7 hours, the appearance was clear and sediment free. Due to the fact that she received this medication by donation, her health insurance plan and her family were unable to pay for this expensive agent, which costs approximately US$14,000 per patient per year in the USA [6]. Although the medical team had a comprehensive discussion with the patient and her family about the likelihood of a decrease in the efficacy of the medication and potential harm, the patient and her parents insisted on using the frozen drugs. Hence, she was closely monitored after injection.

### Outcome and follow-up

**Fig. 1:**
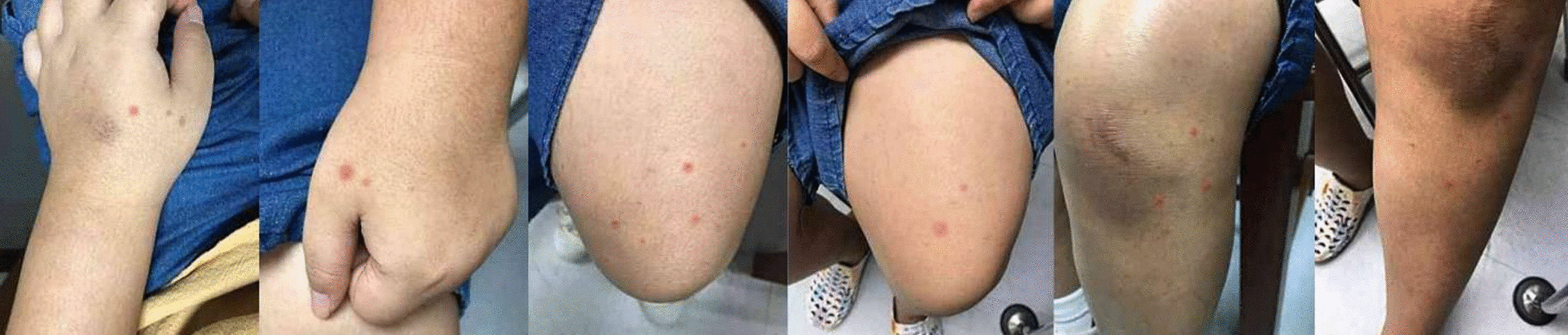
Generalized discrete blanchable to nonblanchable erythematous macules occurred at both upper and lower extremities 14 days after a second dose of frozen drug

Before being injected with the first dose of frozen alirocumab, the patient was generally well. Her blood pressure was 106/56 mmHg, and she weighed 57.2 kg, with a body mass index of 27.1 kg/m^2^. She had a cushingoid appearance from exogenous steroid use but was normal otherwise. After receiving the frozen drug, she did not experience any unusual symptoms and had no reaction at the injection site. The second dose of the frozen drug was administered 2 weeks later. After fourteen days, the patient had generalized discrete blanchable to nonblanchable erythematous macules in her upper and lower extremities, without any skin reaction at the injection site (Fig. 1). After excluding other causes of the rash such as other new drugs including dietary supplements and alternative medications, insect bites, and so on, the dermatologist suspected minor cutaneous drug reaction with a Naranjo score [7] of five, indicating a probable cutaneous drug reaction. As the patient was a minor and the results of the skin biopsy would not change the patient’s management, the patient refused it. The lesion spontaneously resolved without any treatment approximately 1 month later.

**Fig. 2:**
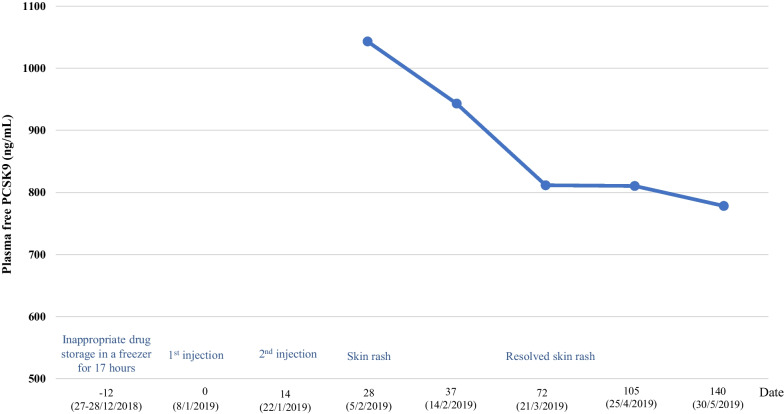
Plasma free PCSK9 after frozen alirocumab injection

Plasma PCSK9 levels were measured using a quantitative enzyme-linked immunosorbent assay (ELISA). The complete methodology of PCSK9 quantification has been described previously [5]. In brief, protein G Agarose and washing buffer were used to remove other immunoglobulin Gs from the serum sample and drug complex before an experiment. The concentration of plasma free PCSK9 was measured using PCSK9 ELISA kits (Human PCSK9 Simple Step ELISA Kit, ABCAM model ab209884). Plasma free PCSK9 concentration was determined by comparing sample luminescence to the reference luminescence curve. The result of plasma free PCSK9 levels after injecting frozen alirocumab is shown in Fig. 2. The lipid profile, severity, and treatment of nephrotic syndrome after injection of frozen alirocumab are presented in Table 1. Serum total cholesterol, free PCSK9, and Lp(a) significantly decreased after the injection. Hence, the drugs still had PCSK9 inhibitory activity even after being inappropriately stored in a freezer for up to 17 hours.

## Discussion and conclusions

We reported on a refractory nephrotic syndrome case with markedly elevated plasma PCSK9 levels. The patient’s PCSK9 and LDL-C plasma levels decreased after PCSK9 mAb injection along with a decrease in the amount of proteinuria. Her plasma PCSK9 level was 1043 ng/mL, while the normal human range of PCSK9 plasma is 170–220 ng/mL [8]. These findings support the idea that elevated PCSK9 expression is a major contributor to the development of hyperlipidemia in NS. PCSK9 activity is increased in individuals with NS and it is closely associated with proteinuria [1]. Therefore, inhibition of PCSK9 activity is an attractive target for treating hypercholesterolemia associated with NS.

PCSK9 mAb significantly lowers plasma LDL-C levels when administered either alone or in combination with statins in hypercholesterolemia associated with refractory nephrotic syndrome [9]. It was interesting to observe a reduction in plasma free PCSK9, and plasma LDL-C levels even after injection of frozen PCSK9 mAb. Nevertheless, the levels of plasma PCSK9 in this patient should be interpreted with caution because the degree of proteinuria is directly associated to the level of hyperlipidemia and PCSK9 [1]. This patient was initiated with PCSK9 mAb treatment, along with a change in the immunosuppressive agent and amount of proteinuria. Therefore, the reduction of plasma PCSK9 and LDL-C levels in this patient may have been influenced by both PCSK9 mAb and severity of proteinuria. Moreover, Lp(a) levels were significantly elevated in NS [10]. The patients also had a considerable reduction of Lp(a) with PCSK9 mAb.

As a therapeutic protein, PCSK9 mAb easily degrades if stored in improper temperatures. The previous study proved that keeping PCSK9 mAb at room temperature in a tropical climate (30.4 ± 2.6 °C) significantly reduced the drug’s effectiveness [5]. Even though PCSK9 mAb has been available for a while, many medical providers may not realize the necessity of properly storing this expensive medication, and thus improper storage may occur. According to our extensive review, this is the first case report to describe the effects of injecting PCSK9 mAb that was frozen and thawed in the refrigerator.

Although its ability to lower plasma LDL-C remains intact, the patient developed a skin lesion 2 weeks after receiving the second dose of frozen drug. According to the ODYSSEY OUTCOMES trial, general allergic reactions occur in up to 7.9% of the alirocumab group compared with 7.8% in the placebo group [11]. However, after the initial injection of two doses of the properly stored drugs and the first dose of the frozen drug, the patient experienced no side effects.  But the patient developed a rash following the second injection of the frozen drug, which can also happen in the case of immune complex or delayed types of drug allergy. This type of drug rash usually occurs within 1–3 weeks after the exposure of suspected drugs [12, 13]. This patient had a Naranjo, adverse drug reaction probability scale, score of 5, indicating a probable cutaneous drug reaction [7]. She had never previously experienced a rash and there were no other new drugs before the rash developed. Frozen drug-induced rash and allergic reactions should be considered. The frozen drug was exposed to a variety of stressors throughout the freezing process, including cold denaturation, freeze concentration, ice crystal formation, and potential excipient crystallization [14]. This rash might have occurred due to the specific protein precipitate in the frozen drug. Nonetheless, the histopathology of the rash was unknown as there was no proven skin biopsy; therefore, the exact etiology of the rash remains unclear.

In conclusion, even though the effectiveness of frozen PCSK9 mAb appears to be stable, it is strongly recommended to keep the medication in proper conditions at all times. Improperly stored drugs should be discarded to avoid any potential undesirable side effects.

## Data Availability

Additional data supporting the findings in this patient are available upon reasonable request.

## Abbreviations

NS: Nephrotic syndrome
PCSK9: Proprotein convertase subtilisin/kexin type 9
mAb: Monoclonal antibody
IDL: Intermediate-density lipoprotein
VLDL: Very low-density lipoprotein
LDL: Low-density lipoprotein
LDL-C: Low-density lipoprotein-cholesterol
Lp(a): Lipoprotein(a)
FSGS: Focal segmental glomerulonephritis
MMF: Mycophenolate mofetil
ELISA: Enzyme-linked immunosorbent assay
